# Oncomir *miR-125b* Suppresses p14^ARF^ to Modulate p53-Dependent and p53-Independent Apoptosis in Prostate Cancer

**DOI:** 10.1371/journal.pone.0061064

**Published:** 2013-04-09

**Authors:** Sumaira Amir, Ai-Hong Ma, Xu-Bao Shi, Lingru Xue, Hsing-Jien Kung, Ralph W. deVere White

**Affiliations:** 1 Department of Urology, University of California Davis, Sacramento, California, United States of America; 2 Department of Biochemistry and Molecular Medicine, School of Medicine, University of California Davis, Sacramento, California, United States of America; Baylor College of Medicine, United States of America

## Abstract

MicroRNAs are a class of naturally occurring small non-coding RNAs that target protein-coding mRNAs at the post-transcriptional level and regulate complex patterns of gene expression. Our previous studies demonstrated that in human prostate cancer the miRNA *miR-125b* is highly expressed, leading to a negative regulation of some tumor suppressor genes. In this study, we further extend our studies by showing that *miR-125b* represses the protein product of the ink4a/ARF locus, p14^ARF^, in two prostate cancer cell lines, LNCaP (wild type-p53) and 22R*v*1 (both wild type and mutant p53), as well as in the PC-346C prostate cancer xenograft model that lentivirally overexpressed *miR-125b*. Our results highlight that *miR-125b* modulates the p53 network by hindering the down-regulation of Mdm2, thereby affecting p53 and its target genes p21 and Puma to a degree sufficient to inhibit apoptosis. Conversely, treatment of prostate cancer cells with an inhibitor of *miR-125b* (anti-*miR-125b*) resulted in increased expression of p14^ARF^, decreased level of Mdm2, and induction of apoptosis. In addition, overexpression of *miR-125b* in p53-deficient PC3 cells induced down-regulation of p14^ARF^, which leads to increased cell proliferation through a p53-independent manner. Thus, we conclude that *miR-125b* acts as an oncogene which regulates p14^ARF^/Mdm2 signaling, stimulating proliferation of prostate cancer cells through a p53-dependent or p53-independent function. This reinforces our belief that *miR-125b* has potential as a therapeutic target for the management of patients with metastatic prostate cancer.

## Introduction

Metastatic prostate cancer (CaP), by progressing to castration-resistant CaP (CRPC), represents a major threat to the life of American men, resulting in estimated 28,170 deaths from this disease in 2012 [1]. Patients with metastatic CaP are customarily treated with androgen deprivation therapy (ADT). Unfortunately, failure of ADT inevitably occurs and the patient's tumor becomes CRPC. It is known that during CRPC progression CaP cells use a variety of androgen receptor (AR)-dependent and independent pathways to survive and flourish in an androgen-depleted environment [2]. Although several attempts have been made to characterize the molecular signature of CRPC, the precise mechanisms leading to CRPC are not completely understood. In recent years, the discovery of microRNAs (miRNAs) has uncovered a new layer of complexity that governs the mechanisms involved in regulating CRPC [3], [4].

MicroRNAs are small non-coding RNAs that function as sequence-specific regulators of gene expression through translational repression and/or transcript cleavage [5]. Studies have shown that miRNAs play key roles in cellular processes of differentiation, proliferation, apoptosis and metabolic homeostasis [6]. Moreover, miRNAs can function as either tumor suppressors or oncogenes, depending on whether they specifically target oncogenes or tumor suppressor genes [7]. In this regard, tumor suppressive miRNAs are usually under-expressed while oncogenic miRNAs tend to be over-expressed in cancer [8]. Studies have shown that *miR-125b* is oncogenic. Overexpression of *miR-125b* was reported in colon cancer [9], bladder cancer [10], ovarian cancer [11] and leukemia [12]. We previously reported that clinical CaP tumors express increased levels of *miR-125b* compared to benign tissues [13]. Additionally, several studies have indicated that *miR-125b* is highly expressed in CaP, particularly in metastatic and invasive CaP tumors [14], [15]. Recently, we investigated the function of *miR-125b* and observed that overexpression of *miR-125b* promoted xenograft tumor growth in both intact and castrated mice [16]. Moreover, we demonstrated that *miR-125b* directly targets several tumor suppressive and proapoptotic genes including p53, Bak1 and Puma [13], [16].

The cellular level and activity of p53 is maintained by a complex circuit comprised of p14^ARF^/Mdm2/p53 [17]. p14^ARF^ was verified to be a potent tumor suppressor both *in vitro* and *in vivo* [18] and has been proposed to be the most important member of this surveillance circuit. Expression of p14^ARF^ is induced in response to activated oncogenes such as Ras [19], c-Myc [20], Abl [21] and E2F-1 [22] as well as during replicative senescence [23]. p14^ARF^ mediates the sequestration and subsequent degradation of the p53-antagonist Mdm2 through the ubiquitin/proteasome pathway, which results in the stabilization (increased half-life) of p53 [17] and the consequent activation of its downstream target genes, such as p21 (cyclin-dependent kinase inhibitor 1A), Puma (p53-upregulated mediator of apoptosis), and Bax (BCL2-associated X protein) [24], [25]. Since these molecules are key components in the p53 network, modulation of their expression can disrupt the normal balance between apoptosis and cell proliferation. This observation is further substantiated by our studies showing that inactivation or down-regulation of p53, Puma and Bak1 by *miR-125b* is associated with CRPC [13], [16].

To further elucidate the role of *miR-125b* in the development of CRPC and its underlying molecular mechanisms, in this study we investigated the involvement of *miR-125b* in modulating the p53 network by targeting p14^ARF^, which is supported by our identification of a potential *miR-125b* binding site in the 3′UTR of *p14^ARF^* gene. We expect our studies to provide new insight into the molecular mechanisms related to tumorigenesis and castration resistant growth of CaP and help in facilitating the application of *miR-125b* as a target for CaP treatment.

## Materials and Methods

### Antibodies and reagents

For Western blotting analysis, anti-p14^ARF^ (sc-8340), anti-Mdm2 (sc-965), were purchased from Santa Cruz Biotechnology (Santa Cruz, CA); anti-Bak1 (3814), anti-Mcl-1(4572), anti-Bcl-X_L_, anti-caspase 3 (9662), anti-SMAC (2954) and anti-p21 (DCS60) were purchased from Cell Signaling Technology (Danvers, MA); anti-Puma (PC686), anti-p53 (OP43) from Calbiochem (Billerica, MA); anti-β-actin (clone AC-15) from Sigma (St. Louis, MO). Synthetic *miR-125b* mimic (miR-125bm), miRNA negative control (miR-NC), anti-*miR-125b* and anti-miRNA negative control (anti-miR-NC) as well as the pMIR-REPORT Luciferase vector were purchased from Ambion (Grand Island, NY). Both *p14ARF* siRNA (sip14) and *Bak1* siRNA (siBak) were purchased from Santa Cruz Biotechnology (Santa Cruz, CA).

### Cell Lines and transfection

Human CaP cell lines PC3, 22R*v*1 and LNCaP were obtained from the American Type Culture Collection (Manassas, VA). All the cell lines were routinely maintained in RPMI 1640 medium supplemented with 10% fetal bovine serum containing antibiotics and multivitamins. For transient transfection, cells were plated onto 6-well plates one day before the transfection and maintained in serum-containing medium without antibiotics. The following day, cells were transfected with either miRNA or siRNA using lipofectamine 2000 (Invitrogen, Grand Island, NY) according to manufacturer instructions.

### Western blot analysis

Cells were grown to 70–80% confluence and lysed using the cell lysis buffer (Cell Signaling Technology) supplemented with phenylmethylsulfonyl fluoride (1 mmol/L). After 20 min of incubation on ice, lysates were centrifuged at 13,000 RPM for 20 min and protein concentrations in the supernatant were determined using BCA kit (Pierce, Rockford, IL). Total protein (50 µg per sample) in 3× protein sample buffer [50 mmol/L Tris-HCl (pH 6.8), 2% SDS, 10% glycerol, 0.25% β-mercaptoethanol, bromophenol blue (1 mg/mL)] were separated on SDS-polyacrylamide gel (Bio-Rad, Hercules, CA), and then transferred to Immobilon PVDF membrane (Millipore, Billerica, MA). After blocking with 5% non-fat dry milk in Tris-buffered saline/0.05% Tween 20 (TBST), the membrane was incubated with a specific primary antibody followed by the horseradish peroxidase-conjugated secondary antibody. Protein bands were displayed by enhanced chemiluminescence. The expression level of protein was measured by quantitative densitometric analysis.

### Luciferase assay

The human *p14^ARF^* 3′-UTR sequence containing the putative *miR-125b* binding site was amplified by PCR from LNCaP cDNA and cloned into the pMIR-REPORT luciferase vector downstream of the luciferase gene. The *p14^ARF^* 3′-UTR lacking this *miR-125b* binding site was used as control. The PCR products cloned into the plasmid were verified by DNA sequencing. For the luciferase assay, cells (4×10^4^ per well) were seeded into 24-well plates and cultured for 24 hrs. The cells were then co-transfected with reporter plasmids and 100 nM synthetic miR-125bm or miR-NC. The pRL-SV40 Renilla luciferase plasmid (Promega, Madison, WI) was used as an internal control. Two days later, cells were harvested and lysed with passive lysis buffer (Promega). Luciferase activity was measured using a dual luciferase reporter assay (Promega). Luciferase activity was normalized by Renilla luciferase activity.

### Co-immunoprecipitation assay

The protein interaction between p14^ARF^ and Mdm2 was detected by co-immunoprecipitation assay. Total protein lysates from miR-125bm- or miR-NC-transfected 22R*v*1 cells were prepared in the cell lysis buffer. Protein (1.0 mg/0.5 ml) was pre-cleared by mixing with 20 µl of protein A beads and the supernatant was immunoprecipitated at 4°C overnight with a rabbit anti-p14^ARF^ polyclonal antibody or normal rabbit IgG (Cell Signaling Technology). The precipitated proteins were fractionated in a 12% SDS-PAGE gel followed by Western blotting detection of Mdm2 protein using the anti-Mdm2 antibody.

### TUNEL assay

TUNEL assay was performed using an *in situ* cell death detection kit (Roche, Indianapolis, IN) according to the manufacturer's instruction. Briefly, p53-positive 22R*v*1 or p53-null PC3 cells (1×10^5^/well) were seeded into individual wells of 4-well chamber slides. After 24 hrs, cells were transfected with 50 nM *miR-125b*, 50 nM anti-*miR-125b* and 100 nM sip14, alone or in different combinations. Untreated and irradiated cells were used as negative and positive controls. Medium was removed 72 hrs after the transfection and slides were rinsed twice with PBS, fixed in a fixation solution (4% paraformaldehyde in PBS, pH 7.4) for 1 hr at RT. After fixation, slides were rinsed twice with PBS and incubated in permeabilization solution (0.1% Triton X-100) for 2 min on ice. 50 µl of the TUNEL reaction mixture (50 µl of enzyme solution+450 µl of label solution) was added to each slide. For the negative control, only 50 µl of the label solution was added. DAPI was used as a nuclear counterstain. Slides were incubated in a humidified atmosphere for 60 min at 37°C in the dark. Fluorescence microscopy was performed to visualize cells and acquire digital images using an excitation wavelength in the range of 450–500 nm and detected in the range of 515–565 nm.

### WST-1 assay

Cells (4.5×10^3^/well) were plated in 96-well plates in RPM1 medium containing 10% FBS. After being cultured for 24 hrs, cells were transfected with 50 nM *miR-125b* or anti-*miR-125b*. After five hrs, cells were treated with fresh medium. Tetrazolium-based cell proliferation assay (WST-1, Promega) was carried out according to the manufacturer's protocol.

### Colony assay

22R*v*1 (3×10^3^/well) and LNCaP (4×10^3^/well) were separately plated in six-well plates and transfected with *miR-125b* or anti-*miR-125b* at a concentration of 100 nM using lipofectamine 2000. After two weeks, cell colonies were counted after staining in 20% methanol and crystal violet.

## Results

### *miR-125b* down-regulates p14^ARF^ in CaP cells

**Figure 1:**
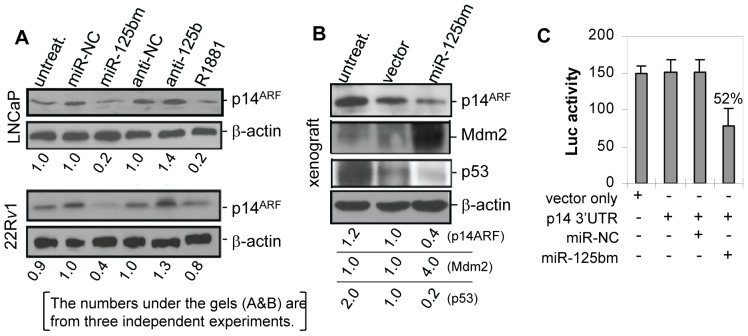
*MiR-125b* down-regulates p14^ARF^ in CaP cells. *A*) Western blot analysis of expression levels of p14^ARF^ in LNCaP (*top*) and 22Rv1 cells (*bottom*). Cells grown in 10% FBS media were transfected with 50 nM of miR-125bm or anti-*miR-125b* (anti-125b) for 72 hrs or treated with 5.0 nM of R1881 androgen for 48 hrs. Then, 50 µg of protein per sample was analyzed. Both miR-negative control (miR-NC) and anti-miR negative control (anti-NC) were used as controls, and β-actin was used as a loading control. *B*) Western blot analysis of expression levels of p14^ARF^, mdm2 and p53 in lenti-*miR-125b*-overexpressed PC-346C xenograft tumor. Both untreated xenograft (untreat.) and lenti-miRNA control vector-infected PC-346C xenograft (vector) were used as controls. In both *A* and *B*, the numbers under the gels are the average fold changes of p14^ARF^ protein from three independent gels relative to the corresponding controls. Fold changes were calculated by scanning the p14^ARF^ bands and normalizing for β-actin bands. *C*) Luciferase assay of *miR-125b* binding to the 3′-UTR of *p14^ARF^* mRNA in LNCaP cells. The assay was repeated three times with each assay being performed in three wells and similar results were obtained each time. The representative results are shown as a mean ±SD (n = 3).

Previous studies demonstrated that the tumor suppressor gene p14^ARF^ is significantly down-regulated in CaP tissues [26]; however, how p14^ARF^ is down-regulated remained poorly understood. Using the TargetScan algorithm, a potential *miR-125b* binding site was identified in the 3-′UTR of *p14^ARF^* mRNA. We thus investigated the effect of *miR-125b* on the regulation of p14^ARF^ in CaP cells. To do this, LNCaP and 22R*v*1 cells were transfected with synthetic miR-125bm to elevate the cellular *miR-125b* abundance, or with anti-*miR-125b* to repress *miR-125b* activity. As shown by Western blot and quantitative densitometric analyses, compared to the miR-NC treatment, miR-125bm induced reduction of p14^ARF^ expression by 80% in LNCaP cells (Figure 1A, top panel) and 60% in 22R*v*1 (Figure 1A, bottom panel). Conversely, anti-*miR-125b* increased the p14^ARF^ level by 40% in LNCaP (Figure 1A, top panel) and 30% in 22R*v*1 (Figure 1A, bottom panel) compared to anti-miR-NC. Our previous study demonstrated that androgen up-regulates *miR-125b* in CaP cells [13]. Thus, LNCaP and 22R*v*1 cells were treated with 5.0 nM of R1881 androgen and the expression level of p14^ARF^ was determined. It was found that R1881 treatment induced an 80% reduction of p14^ARF^ in LNCaP and 20% decrease in 22R*v*1 (Figures 1A). We also examined the level of p14^ARF^ in a *miR-125b*-overexpressed PC-346C mouse xenograft tumor [16], and found that the level of p14^ARF^ protein was reduced by 60% in the *miR-125b*-overexpressed tumor compared to miR-NC control tumor (Figure 1B). To determine whether the putative *miR-125b* binding site in the 3′-UTR of p14^ARF^ mRNA is responsible for the regulation of p14^ARF^ by *miR-125b*, luciferase reporter vectors containing the 3′-UTR fragment of *p14^ARF^* gene were co-transfected with miR-125bm into LNCaP cells. As shown in Figure 1C, cotransfection resulted in an approximately 50% reduction of the enzyme activity in LNCaP cells. We also performed luciferase assay in 22R*v*1 cells and a similar result was observed (data not shown). Taken together, the results shown in Figure 1 validate the regulation of p14^ARF^ by *miR-125b* in CaP cells.

### *miR-125b*-p14^ARF^signaling regulates the p53 network

**Figure 2:**
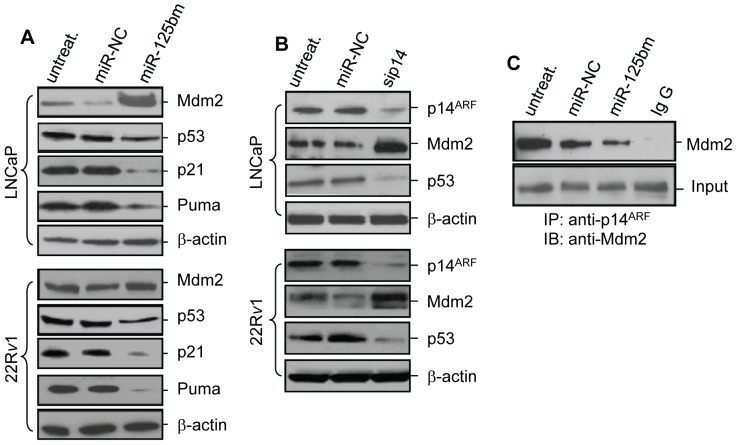
*MiR-125b* regulates the p53 network. *A*) Western blot analysis of Mdm2 and p53 in miR-125bm-treated LNCaP (*top*) and 22R*v*1 cells (*bottom*). Cells were transfected with 50 nM of miR-125bm or miR-negative control (miR-NC) for 72 hrs. Equal amounts of protein (50 µg) were used to detect the expression levels of Mdm2, p53, p21 and Puma. *B*) Western blot analysis of p14^ARF^, Mdm2 and p53 in *p14^ARF^* siRNA (sip14)-treated LNCaP (*top*) and 22R*v*1 cells (*bottom*). Cells were treated with sip14 and the cellular levels of p14^ARF^, p53 and Mdm2 were analyzed. β-actin was used as a loading control. *C*) Co-immunoprecipitation analysis of protein interaction between p14^ARF^ and Mdm2 in 22R*v*1 cells. Cells were transfected with miR-125bm and 1.0 mg protein was immunoprecipitated with anti-p14^ARF^ antibody or the rabbit IgG. The resultant immunecomplexes were used to detect the level of Mdm2 by Western blot analysis using anti-Mdm2 antibody. Input: 50 µg protein from total cell lysate. IP: immunoprecipitation. IB: immunoblotting.

Studies have established that p14^ARF^ accelerates Mdm2 degradation, resulting in p53 up-regulation [27]. We thus asked: does down-regulation of p14^ARF^ by *miR-125b* affect the expression of Mdm2 and p53 in CaP cells? To address this issue, LNCaP and 22R*v*1 cells were treated with miR-125bm and the levels of Mdm2 and p53 were then examined. Compared with miR-NC, treating LNCaP cells with *miR-125b* induced a dramatic increase in Mdm2 expression and a significant reduction of p53 level (Figure 2A, top panel). Similarly, in 22R*v*1 cells, *miR-125b* treatment also enhanced Mdm2 expression and reduced p53 level (Figure 2A, bottom panel). As expected, miR-125bm-mediated down-regulation of p53 induced significant reduction of two direct p53 effectors, p21 and Puma. Similarly, in the *miR-125b*-overexpressed PC-346C xenograft tumor, Mdm2 expression was increased three-fold and p53 protein was down-regulated by 83% when compared to the vector control (Figure 1B). To confirm the downstream results from inhibition of p14^ARF^, we used *p14^ARF^* siRNA (sip14) to silence p14^ARF^ in LNCaP and 22R*v*1 cells. As shown by immunoblotting, sip14 treatment significantly decreased the expression of p14^ARF^ protein and subsequently upregulated Mdm2 level and downregulated the expression of p53 (Figure 2B). Since p14^ARF^ directly binds to the C-terminal of Mdm2, we examined the effect of *miR-125b* on the protein interaction between p14^ARF^ and Mdm2 by co-immunoprecipitation in 22R*v*1 CaP cells. We observed that Mdm2 can be detected from anti-p14^ARF^ antibody-precipitated proteins, not from control IgG-coupled proteins, indicating that endogenous p14^ARF^ is capable of forming a complex with Mdm2. Treatment with *miR-125b* down-regulated p14^ARF^protein, resulting in a reduction of immunoprecipitated Mdm2 (Figure 2C). Taken together, data shown in Figure 2 provide evidence that *miR-125b* regulates p14^ARF^/Mdm2/p53 signaling pathway.

### *miR-125b* stimulates proliferation of CaP cells

**Figure 3:**
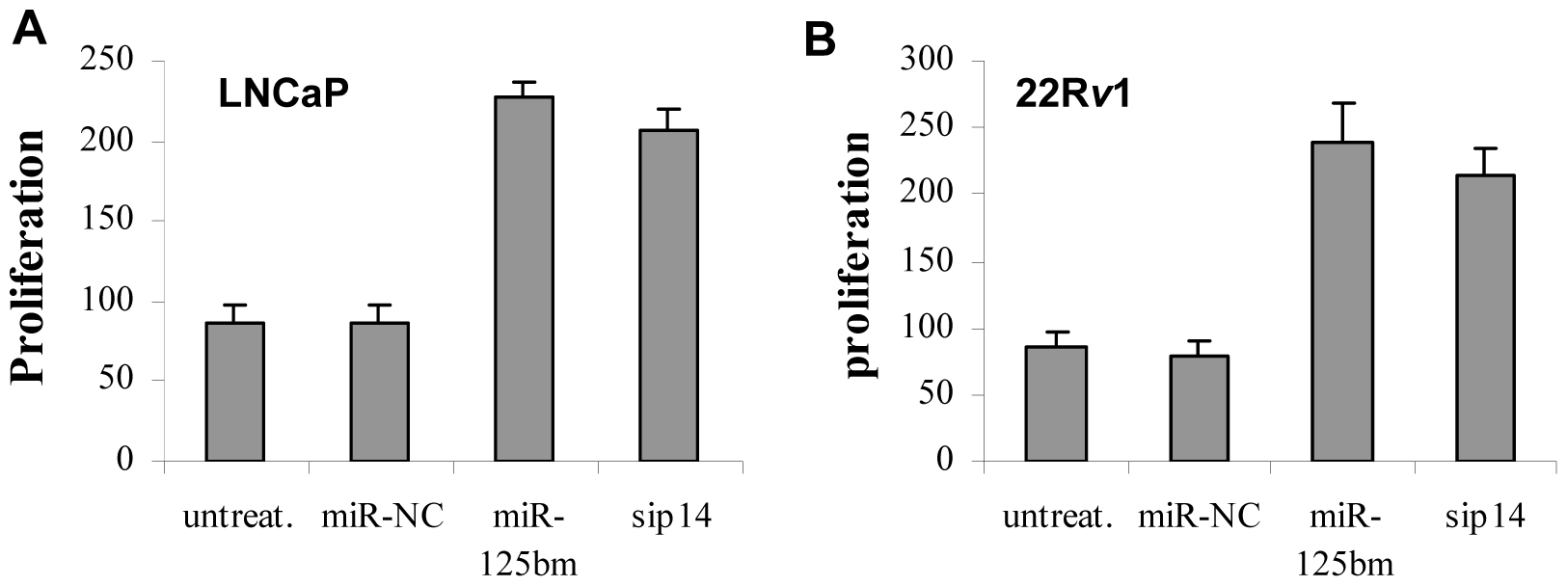
WST-1 proliferation assay of LNCaP cells (*A*) and 22R*v*1 cells (*B*). Cells were transfected with 50 nM of miR-125bm or 50 nM of miRNA negative control (miR-NC) for 5 days. Cell proliferation was measured by WST-1 assay. *p14^ARF^* siRNA (sip14) was used as a control. The results are expressed as proliferation relative to that of miR-NC-treated cells, and shown as mean ± SD (n = 4).

Having determined the regulation of p14^ARF^/Mdm2/p53 signaling pathway by *miR-125b*, we next examined the effect of regulation of p14^ARF^ by *miR-125b* on CaP cell proliferation. To do this, both LNCaP cells and 22R*v*1 cells were transfected with synthetic miR-125bm and cell proliferation was determined by WST-1 assay. As shown in Figures 3A and 3B, when compared with the miR-NC treatment, transfection with miR-125bm resulted in a 1.5-fold increase in cell proliferation in both cell lines tested. In addition, we performed clone formation assays. Similar to the WST-1 results, *miR-125b* stimulated a 1.0-fold increase in clonogenic survival of LNCaP cells and 2.5-fold enhancement in 22R*v*1 cells, and the addition of anti-*miR-125b* caused a dramatic reduction in the number of colonies as compared to the untreated and anti-miR-NC cells (data not shown). These data support that downregulation of p14^ARF^ by *miR-125b* facilitates growth of CaP cells.

### Anti-*miR-125b* induced apoptosis in CaP cells expressing functional p53

**Figure 4:**
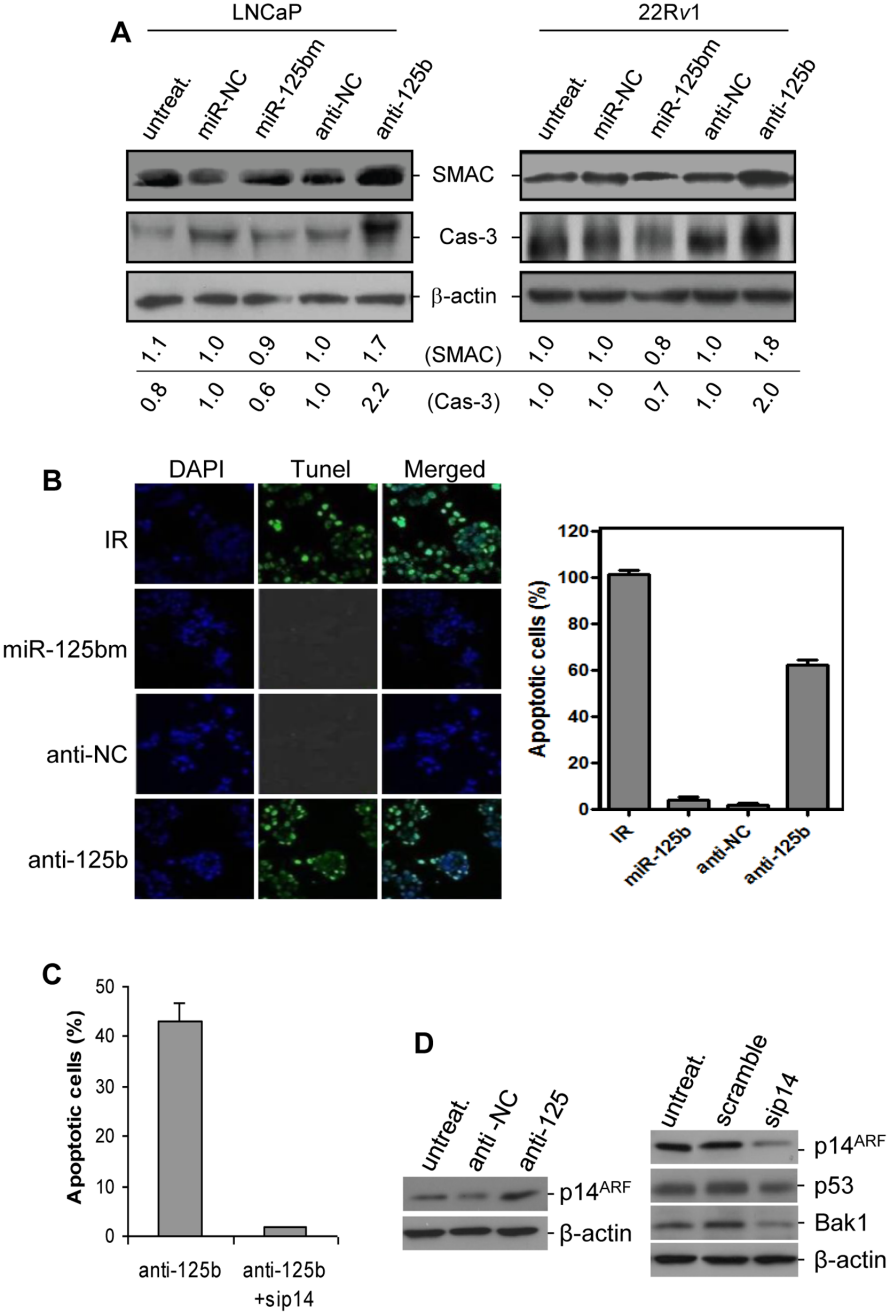
Inactivation of *miR-125b* induces apoptosis in p53-positive CaP cells. *A*) Detection of SMAC and activated caspase 3 (Cas-3) in LNCaP (*left*) and 22R*v*1 (*right*) cells. Cells were transfected with 50 nM miR-125bm or 50 nM anti-*miR-125b* (anti-125b) for 5 days, and the levels of SMAC and Cas-3 were measured by Western blot analysis. β-actin was used as loading control. The numbers under the gels are the average fold changes of SMAC and Cas-3 from three independent gels relative to the corresponding controls. *B*) Detection of anti-*miR-125b*-induced apoptosis in 22R*v*1 cells. Cells were transfected using 50 nM anti-*miR-125b* for 72 hrs and apoptotic cell death was detected using TUNEL assay. The green nuclear fluorescence indicates the apoptotic cleavage of nuclear DNA (*left*). For quantitation of apoptotic cell death, 400 cells were counted and apoptosis is expressed as % apoptosis (apoptotic cells/400×100%). Quantitative analysis was performed three times and result was expressed as mean ± SE (n = 3) (*right*). Cells treated with irradiation (IR, 6 Gy) were used as a positive control. *C*) TUNEL assay of apoptotic death of 22R*v*1 cells that were treated with anti-*miR-125b* followed by *p14^ARF^* antisense (sip14). Result was expressed as mean ± SE (n = 3). *D*) Western blot analyses of p14^ARF^, p53 and Bak1 levels in 22R*v*1 cells. *Left*: 22R*v*1 cells were transfected with anti-*miR-125*; *right*: anti-*miR-125*-transfected 22R*v*1 cells were treated with sip14. Both anti-miR-NC (anti-NC) and scramble siRNA were used as controls.

Since *miR-125b* regulates p14^ARF^/Mdm2 signaling and subsequently affects the p53 network, we evaluated the effect of downregulation of p14^ARF^ by *miR-125b* on apoptosis in p53-positive CaP cells. First, we tested the release of mitochondrial SMAC (second mitochondria-derived activator of caspase) and activated caspase 3 (Cas-3) in LNCaP and 22R*v*1 cell lines that express functional p53. When compared to miR-NC treatment, miR-125bm caused 10% reduction of SMAC and 40% reduction of activated Cas-3 in LNCaP cells, and the reduction was 20% and 30% in 22R*v*1 cells, respectively (Figure 4A). These cell lines were also treated with anti-*miR-125b*. Compared to anti-miR-NC treatment, downregulation of *miR-125b* activity induced approximately one-fold increase in SMAC and activated Cas-3 (Figure 4A). Since anti-*miR-125b* upregulates SMAC and activated caspase 3, we thus analyzed anti-*miR-125b*-induced apoptotic cell death by using a TUNEL assay. 22R*v*1 cells were transfected with miR-125bm or anti-*miR-125b*. No apoptotic cell death was observed in miR-125bm-treated 22R*v*1 cells. In contrast, treatment of 22R*v*1 cells with anti-*miR-125b* caused 63% of cells to undergo apoptosis (Figure 4B). To validate that *miR-125b* modulates p53-dependent apoptosis through p14^ARF^, 22R*v*1 cells were treated with anti-*miR-125b*, followed by *p14^ARF^* silencing. It was found that antisense to *p14^ARF^* (sip14) dramatically decreased apoptotic death in *miR-125b*-inactivated 22R*v*1 cells (Figure 4C). As expected, *p14^ARF^* silencing stimulated proliferation of these 22R*v*1 cells (data not shown). In addition, the expression levels of several pro-apoptotic factors were assessed with Western blot analysis. Indeed, treatment with anti-*miR-125b* induced an upregulation of p14^ARF^ protein in 22R*v*1 cells, while addition of sip14 resulted in obvious downregulation of p14^ARF^ (60%), p53 (30%) and Bak1 (70%), compared to the scramble siRNA treatment (Figure 4D). These data strongly suggest that *miR-125b*/p14^ARF^ signaling targets the p53 network, regulating p53-dependent proliferation and apoptosis in CaP cells.

### *miR-125b*/p14^ARF^ signaling mediates p53-independent growth inhibition

**Figure 5:**
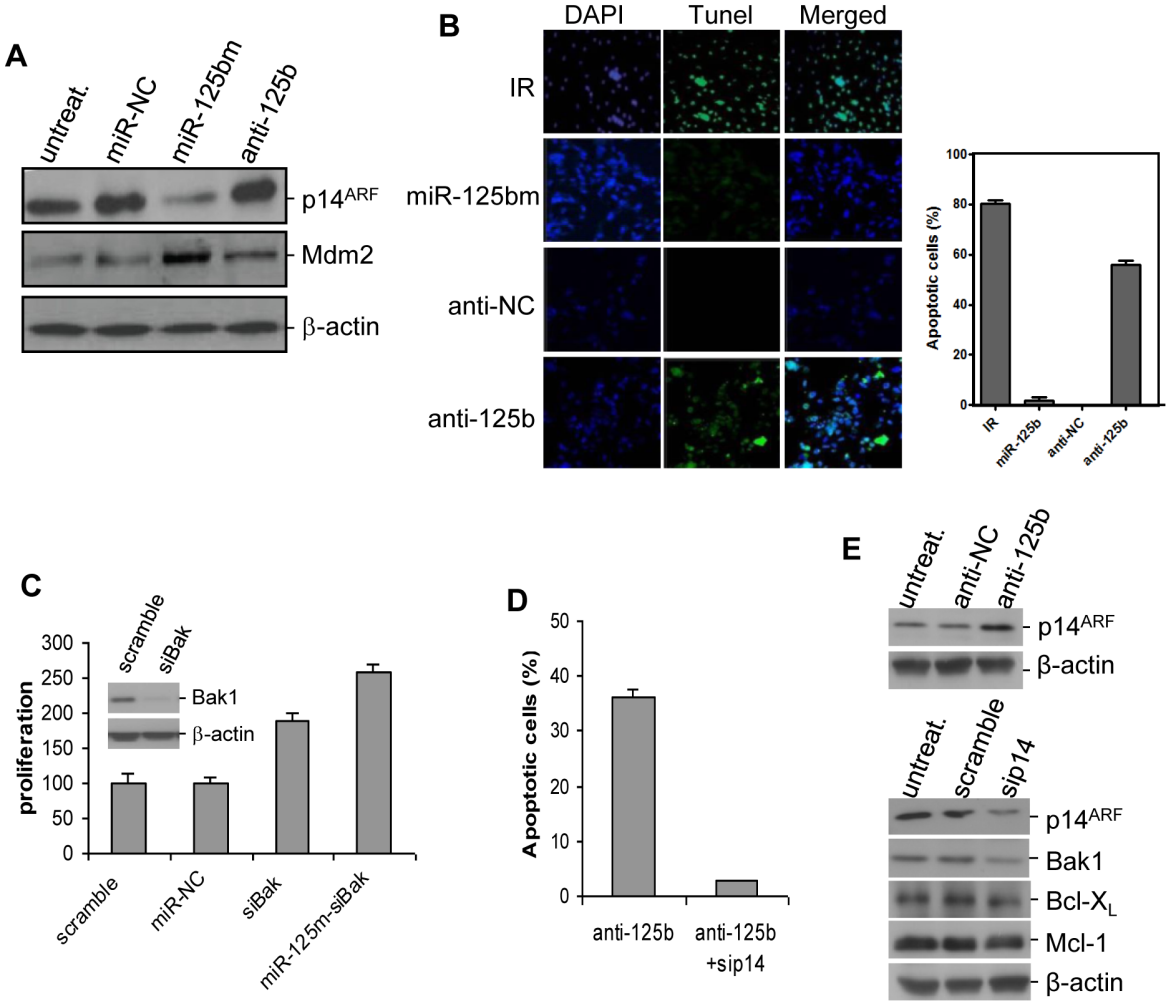
Evaluation of *miR-125b* effect on growth and apoptosis in p53-negative CaP cells. *A*) Detection of p14^ARF^ and Mdm2 levels in p53-null PC3 cells. Cells were transfected with 50 nM of miR-125bm or anti-*miR-125b* for 72 hrs. The expression levels of both p14^ARF^ and Mdm2 were analyzed by Western blot assay. β-actin was used as a loading control. *B*) Detection of anti-*miR-125b*-induced apoptosis in PC3 cells. Cells were transfected using 50 nM anti-*miR-125b* for 72 hrs and apoptotic cell death was detected using TUNEL assay. The green nuclear fluorescence indicates the apoptotic cleavage of nuclear DNA (*left*). For quantitation of apoptotic cell death, 400 cells were counted and apoptosis is expressed as % apoptosis (apoptotic cells/400×100%). Quantitative analysis was performed three times and result was expressed as mean ± SE (n = 3) (*right*). Cells treated with irradiation (IR, 6 Gy) were used as a positive control. *C*) *MiR-125b* promotes the growth of p53-null, *Bak1*-silenced PC3 cells. Cells were treated with 50 nM miR-125m for 5 days and cell proliferation was measured using WST-1 assay. The results are expressed as the growth inhibition relative to that of miR-NC (mean ± SD, n = 4). Inset: Bak1 expression in *Bak1*-silenced PC3 cells. *D*) TUNEL assay of apoptotic death of PC3 cells that were treated with anti-*miR-125b* followed by *p14^ARF^* antisense (sip14). Result was expressed as mean ± SE (n = 3). *E*) Western blot analyses of p14^ARF^ and Bak1 levels in PC3 cells. *Top*: PC3 cells were transfected with anti-*miR-125*; *bottom*: anti-*miR-125*-transfected PC3 cells were treated with sip14. Both anti-miR-NC (anti-NC) and scramble siRNA were used as controls.

In the above experiments, we validated that *miR-125b*/p14^ARF^ signaling is involved in p53-dependent mechanisms in CaP cells. However, studies demonstrated that inactivation of p53 function occurs in a portion of patients with metastatic CaP [28], [29]. Does *miR-125b*/p14^ARF^ signaling regulate cell growth and apoptosis in these p53-deficient CaPs? We used p53-null PC3 CaP cells to address this issue. We examined the influence of altered *miR-125b* activity on the expression levels of p14^ARF^ and Mdm2 proteins. Similar to that in p53-functional LNCaP and 22R*v*1 cells, miR-125bm transfection decreased expression of p14^ARF^ by 36% and increased Mdm2 by 43% in PC3 cells while anti-*miR-125b* induce an obvious upregulation of p14^ARF^ and a slight repression of Mdm2 (Figure 5A). We next tested whether *miR-125b* affects the proliferation and apoptosis of PC3 cells. To this end, PC3 cells were treated with anti-*miR-125b* and apoptotic cells was detected with the TUNEL assay. It was found that treatment with anti-*miR-125b* caused 50% of these cells to undergo apoptosis (Figure 5B). Since Bak1 was reported to mediate p14^ARF^-induced apoptosis in p53-deficient cells [30], we evaluated the effect of *Bak1* silencing on proliferation of miR-125bm-transfected PC3 cells. It was found that miR-125bm induced a 1.6-fold increase in survival of these PC3 cells (Figure 5C), supporting previous observation that p14^ARF^/Mdm2 signaling contributes to a p53-independent mechanism [31]. To confirm the regulation of p53-independent apoptosis by *miR-125b*/p14^ARF^ signaling, *miR-125b* activity was suppressed with anti-*miR-125b* and *p14^ARF^* was silenced by RNAi. We observed that *p14^ARF^* silencing significantly decreased apoptotic death of *miR-125b*-inactivated PC3 cells (Figure 5D), and also stimulated their proliferation (data not shown). Additionally, the expression levels of p14^ARF^ and Bak1 were analyzed. It was found that *miR-125b* inactivation induced an upregulation of p14^ARF^, while *p14^ARF^* silencing reversed the upregulation of p14^ARF^ (60%) and also induced a downregulation of Bak1 (Figure 5E). A previous study reported that both Bcl-X_L_ and Mcl-1 mediate p14^ARF^-induced p53-independent apoptosis. These two anti-apoptotic factors were thus analyzed. We did not observe their alteration in *miR-125b*-inactivated, *p14^ARF^*-silenced PC3 cells (Figure 5D). Taken together, these data show that *miR-125b*/p14^ARF^ signaling is able to regulate growth and apoptosis in p53-deficient CaP cells.

## Discussion

Recent observations of aberrant miRNA expression in various human cancers have highlighted the importance of miRNAs in many biological processes [5]. *MiR-125b* is a broadly conserved miRNA and was found to be elevated in several types of cancers including CaP [14], [15]. We previously reported that clinical CaPs with high Gleason scores highly express *miR-125b* [13], and that *miR-125b* directly targets p53, Puma and Bak1, showing an anti-apoptotic effect in the presence and absence of androgens [16]. Additionally, we observed that *miR-125b* promotes tumor formation and castration resistant growth of CaP cells [16]. In this study, we identified *miR-125b* as a direct negative regulator of p14^ARF^. Our study validated that *miR-125b* can directly repress the p14^ARF^ protein expression through its interaction with the binding site in the 3′-UTR of the human *p14^ARF^* mRNA, thereby inhibiting p14^ARF^ function in CaP cells. Moreover, we observed that *miR-125b* inhibits interaction between p14^ARF^ and Mdm2, with the downstream consequence of modulating the p53 network. Our report is the first to identify *miR-125b* as a direct regulator of p14^ARF^ in CaP cells. Our data showed that the negative regulation of p14^ARF^ by *miR-125b* is physiologically relevant to cellular function, as an increase in *miR-125b* level stimulates cell proliferation and represses intrinsic apoptosis both in androgen-dependent LNCaP cells and CRPC 22R*v*1 cells. The point is underscored by the fact that increasing *miR-125b* in LNCaP cells results in an 80% reduction in p14^ARF^, while the reduction is 60% in 22R*v*1 CRPC cells; when *miR-125b* is elevated through treatment of these cells with R1881, the reduction of p14^ARF^ in LNCaP again is 80%, while it is 20% in 22R*v*1 cells. Additionally, when the reverse is carried out by using anti-*miR-125b* to counter the activity of endogenous *miR-125b* in the two CaP cell lines, the increase in p14^ARF^ is 40% and 30%, respectively. Thus, the downregulation of p14^ARF^ by overexpressed *miR-125b* and subsequent repression of p53 activity are involved in prostatic tumorigenesis and progression.

The tumor suppressor p53 is an important transcription factor that safeguards the cell against tumorigenesis by maintaining a fine balance between apoptosis and cell proliferation [32]. Increasing evidence has shown that the p14^ARF^/Mdm2/p53 pathway is essential for maintaining and regulating p53 expression and function, and an alteration of components in the pathway, like downregulation of p14^ARF^ or upregulation of Mdm2, can significantly alter p53 intracellular level and activity [33]. In this study, we found that *miR-125b* targets p14^ARF^ not only in *miR-125b*-transfected CaP cell lines but also in the *miR-125b*-overexpressed PC-346C xenograft tumor. Therefore, we believe that overexpression of *miR-125b* results in deregulation of the p14^ARF^/Mdm2/p53 pathway, disrupting the balance between apoptosis and cell proliferation. The deregulation of p14^ARF^/Mdm2/p53 pathway by aberrantly expressed *miR-125b* provides a mechanistic explanation for our previous observation that *miR-125b* facilitates tumor formation and castration resistant growth of PC-346C xenograft tumor [16]. Indeed, when the PC-346C xenograft tumor was analyzed for the expression of the components in the p14^ARF^/Mdm2/p53 pathway, we found that overexpression of *miR-125b* resulted in a 60% reduction of p14^ARF^, a 3-fold increase in Mdm2, and an 83% reduction of p53.

**Figure 6:**
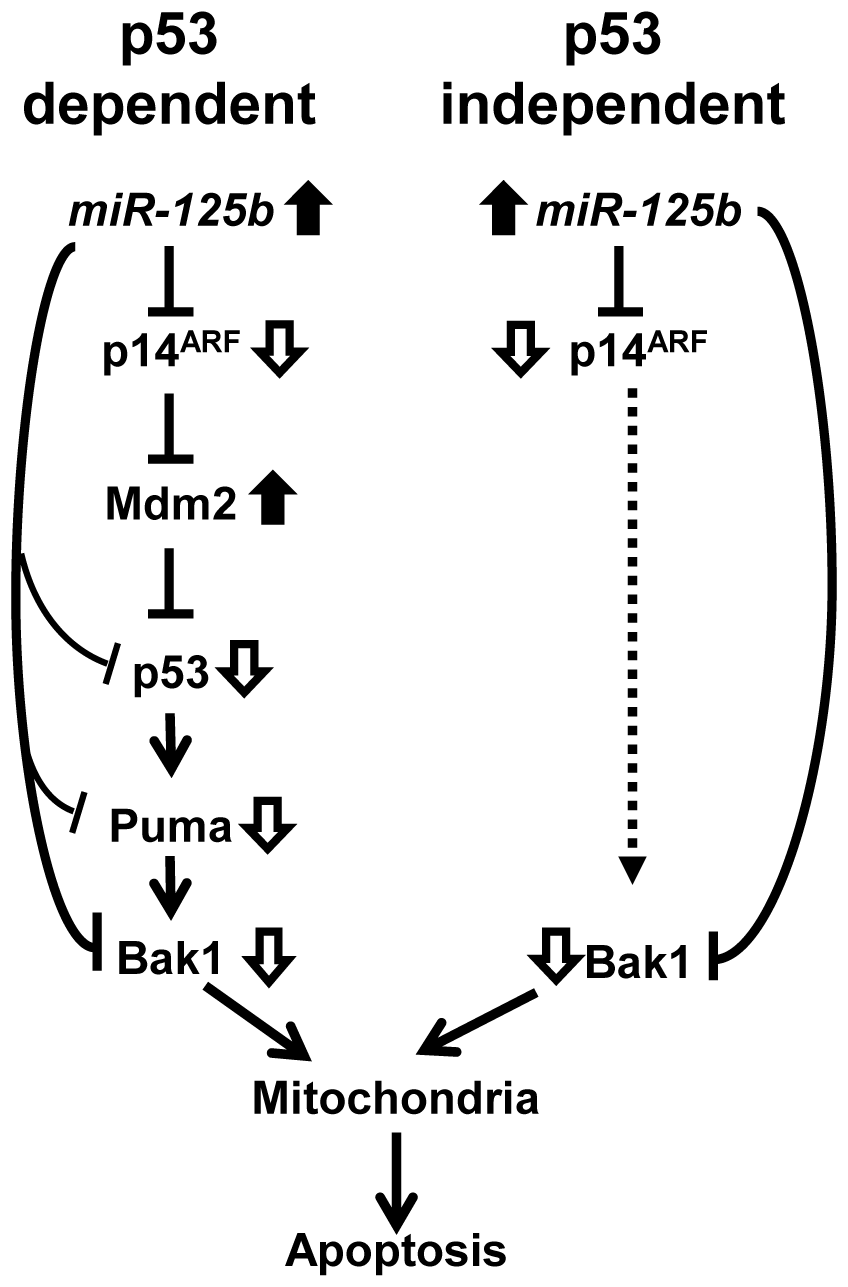
Schematic model of *miR-125b*-controlled oncopathway in CaP cells. In CaP cancer cells, p14^ARF^ facilitates apoptosis in a p53-dependent (*left*) and p53-independent (*right*) manner [30]. Since *miR-125b* directly targets p14^ARF^ and other pro-apoptotic molecules, deregulation of *miR-125b* can modulate proliferation and apoptosis in both p53-positive and p53-deficient CaP cells. Black arrows represent upregulated molecules and white arrows represent downregulated molecules. Broken arrow indicates undefined upregulation of Bak1 activity by p14^ARF^.

If modulation of tumor growth and apoptosis by *miR-125b* was p53-dependent, this would limit the number of patients with metastatic CaP in whom such modulation would be viewed as a therapeutic strategy, because a number of these patients' tumors have defective p53 functions. We previously reported that 10 of 17 (59%) metastatic CaPs obtained before ADT treatment were p53 defective and this rose to 80% in samples obtained after ADT [34]. These findings are in agreement with multiple other reports [29], [35], [36]. Significantly, in this study, we showed that increased level of *miR-125b* modulated p14^ARF^ in p53-null PC3 CaP cells. While we show the functional mechanism of how this occurs in p53-dependent cases, how *miR-125b* regulates proliferation and apoptosis in p53-deficient CaPs has not been clearly defined. Recently, Muer found that p14^ARF^ induces apoptosis in cancer cells in both p53-dependent and p53-independent fashions [30]. Using the data presented in this study and in our previous publications [13], [16], we re-built Muer's pathway (Figure 6). We show that the control of p14^ARF^ is in effect through downregulation by *miR-125b*. However, data provided by Muer showed that p14^ARF^ induces p53-independent apoptosis by inhibition of Mcl-1 and Bcl-X_L_, resulting in activation of Bak1 [30]. We did not observe altered levels of Mcl-1 and Bcl-X_L_ but Bak1 indeed was downregulated in p14^ARF^-silencd PC3 cells. Our data suggest that other molecules may mediate the regulation of Bak1 by p14^ARF^. Additionally, we have previously shown that *miR-125b* has a second control mechanism in both the p53-dependent and p53-independent arms by direct downregulation of p53, Puma and Bak1 in the p53-dependent pathway and by blocking Bak1 in the p53-independent pathway [16]. Thus, this study, taken with our previous published work, supports our belief that *miR-125b* is a potentially important therapeutic target for patients with metastatic CaP.

In the last decade, considerable new molecular information has underlined the mechanisms of response and resistance of metastatic CaP to different interventions. The body of work has led to FDA approval of five new therapies for CRPC (docetaxel, cabazitaxel, Provenge, abiraterone acetate, and MDV3100). Regrettably, they each improve survival by only approximately four to five months [37], [38]. The latter two agents, abiraterone acetate and MDV3100, underscore that while the AR is critical to the process of controlling CaP, targeting it alone will not be sufficient. We believe that the data presented in this paper and in our previous publications [13], [16] offer hope that lowering *miR-125b* in patients with metastatic CaP will attack not a single pathway, but a complicated oncopathway. Modulation of the oncopathway will be both a treatment in itself as well as augmenting presently used interventions. Our ongoing studies are aimed at proving this hypothesis.

In summary, we observed that overexpression of *miR-125b* negatively regulates the expression of the tumor suppressor protein p14^ARF^ and aberrant expression of *miR-125b* promotes cell proliferation potential and inhibits apoptosis. Interestingly, inactivation of *miR-125b* using anti-*miR-125b* affects apoptosis involving both p53-dependent and p53-independent pathways. Therefore, our data presented in this study suggest that oncomir *miR-125b* has a great potential in the design of combination therapy for CaP treatment.
